# The impact of dietary isoflavonoids on malignant brain tumors

**DOI:** 10.1002/cam4.265

**Published:** 2014-06-04

**Authors:** Tina Sehm, Zheng Fan, Ruth Weiss, Marc Schwarz, Tobias Engelhorn, Nirjhar Hore, Arnd Doerfler, Michael Buchfelder, IIker Y Eyüpoglu, Nic E Savaskan

**Affiliations:** 1 Department of Neurosurgery, Erlangen University Medical School, Friedrich Alexander University Erlangen-Nuremberg (FAU)Schwabachanlage 6 (Kopfklinik), D-91054, Erlangen, Germany; 2 Department of Neuroradiology, Erlangen University Medical School, Friedrich Alexander University Erlangen-Nuremberg (FAU)Schwabachanlage 6 (Kopfklinik), D-91054, Erlangen, Germany

**Keywords:** Angiogenesis, experimental therapeutics, microenvironment, nutrition

## Abstract

Poor prognosis and limited therapeutic options render malignant brain tumors one of the most devastating diseases in clinical medicine. Current treatment strategies attempt to expand the therapeutic repertoire through the use of multimodal treatment regimens. It is here that dietary fibers have been recently recognized as a supportive natural therapy in augmenting the body's response to tumor growth. Here, we investigated the impact of isoflavonoids on primary brain tumor cells. First, we treated glioma cell lines and primary astrocytes with various isoflavonoids and phytoestrogens. Cell viability in a dose-dependent manner was measured for biochanin A (BCA), genistein (GST), and secoisolariciresinol diglucoside (SDG). Dose–response action for the different isoflavonoids showed that BCA is highly effective on glioma cells and nontoxic for normal differentiated brain tissues. We further investigated BCA in ex vivo and in vivo experimentations. Organotypic brain slice cultures were performed and treated with BCA. For in vivo experiments, BCA was intraperitoneal injected in tumor-implanted Fisher rats. Tumor size and edema were measured and quantified by magnetic resonance imaging (MRI) scans. In vascular organotypic glioma brain slice cultures (VOGIM) we found that BCA operates antiangiogenic and neuroprotective. In vivo MRI scans demonstrated that administered BCA as a monotherapy was effective in reducing significantly tumor-induced brain edema and showed a trend for prolonged survival. Our results revealed that dietary isoflavonoids, in particular BCA, execute toxicity toward glioma cells, antiangiogenic, and coevally neuroprotective properties, and therefore augment the range of state-of-the-art multimodal treatment approach.

## Introduction

Malignant gliomas (WHO grade III and IV formerly named glioblastoma multiforme) are one of the most common primary brain tumor entities with an annual incidence rate of 5.8 in the United States and in Europe 1,2 The prognosis remains poor despite recent advances in multimodal treatment strategies including antiangiogenic drugs 1,3,4. Severe complications associated with malignant gliomas include vasogenic and cytotoxic brain edema as well as neurodegeneration and necrosis 5–8. A major cause of mortality in more than 60% of patients suffering from glioblastoma is due to cerebral herniation as a consequence of massive increase in intracranial pressure caused by uncontrolled brain edema 9,10. Furthermore, cerebral edema leads to neurological deterioration and hence significant reduction in quality of life. Inhibition of brain edema therefore plays a vital role in the management of malignant gliomas. So far, edema treatment in tumor patients is most commonly treated with dexamethasone 11, a synthetic glucocorticoid with potent anti-inflammatory activity. Most patients undergoing radiochemotherapy receive dexamethasone as a supportive treatment. The significant decrease in deaths related to this treatment scheme indicates the general importance of adjuvant therapeutic approaches and its impact on the course of disease 12.

Despite this, only few attempts have been made in search for nonsteroid-based alternatives in the management of cerebral edema. One proposal would be caloric restriction in influencing tumor metabolism, edema formation, and angiogenesis 13–16. This is often carried out by increasing the dietary component of isoflavonoids, which constitute an integral part of the human diet 17–19. Moreover, isoflavonoids such as biochanin A (BCA) and genistein (GST) have been found to exert positive influence on the inhibition of cancer induction and progression in vitro and in vivo 20–22. Thus, the contribution of caloric restriction and dietary isoflavonoids is not well discriminated in those settings. Isoflavonoids are a large group of polyphenolic compounds occurring in plants 23. These compounds are enriched in vegetables, fruits, and in red clover 24 and their beneficial effects are high in various chronic diseases as well as association with reduction in the general risk of developing cancer is well documented 25,26. The molecular mechanism of action of isoflavonoids is based on compound-specific inhibitory effects on protein tyrosine kinases, poly(ADP-ribose) polymerase cleavage, and caspase-3 activation in the case of GST 27,28, and inhibition of glioma-derived matrix metalloproteinases and phosphorylation of Akt and eIF4E in the case of BCA 29,30.

Here, we examined the effect of the isoflavonoid biochanin A (BCA) from the red clover (trifolium pratence), GST, and the flaxseed component secoisolariciresinol diglycoside (SDG) on nontransformed, that is, physiologic astrocytes and glioma cells. We identified BCA to be highly effective on human and rodent glioma cells while exhibiting a low-toxic profile on healthy tissue. Biochanin A can be regarded as safe with respect to adverse reaction as it does not induce cell death in healthy brain tissue. Hence, BCA prevents spontaneous neuronal damage and exerts antiangiogenic and brain swelling preventive effects in vivo. These results indicate that isoflavonoids exert a variable toxic profile and show beneficial effects in addition to established chemotherapeutics.

## Material and Methods

### Cell culture

Rodent glioma cell line F98, the human glioma cell line U87, and the murine glioma cell line GL261 were obtained from ATCC/LGC (Wesel, Germany). The human glioma cell line U251 was provided by Yvonne Ruebner and Rainer Fietkau (Department of Radiation Therapy, Erlangen University Hospital). Primary rat astrocytes were prepared from up to 1-month-old Wistar rats. All cells were cultured under standard humidification conditions (37°C, 5% CO_2_) with Dulbecco's modified Eagle's medium (DMEM; Biochrom, Berlin, Germany) supplemented by 10% fetal bovine serum (Biochrom), 1% penicillin/streptomycin (Biochrom), and 1% glutamax (Gibco/Invitrogen, Darmstadt, Germany). Cells were passaged at ∼80% confluence. Cells were scrapped of or trypsinized after the phosphate buffered saline (PBS) wash step. Cells were plated out in a culture flask after centrifugation (900 rpm for 5 min).

### Primary astrocytes culture

Primary astrocytes were isolated from rat brains postnatal day 4 to 6 excluding the cerebellum (Charles River Laboratories, Wilmington, MA). Brains freed of meninges were placed in ice-cold Hank's Balanced Salt Solution (HBSS) buffer without serum. Brains were further gently triturated with fire-polished Pasteur pipettes of diminishing tip diameter until tissue was uniformly homogenized. Minced brain tissue was trypsinized with 0.25% trypsin for 10 min. After resuspending and centrifugation, astrocytes were cultured in full DMEM medium (Biochrom) supplemented with 10% fetal calf serum and penicillin/streptomycin (Biochrom). From the following day on astrocyte culture flasks were agitated to separate detaching microglia.

### Chemicals

BCA was purchased from Selleckchem (Absource Diagnostics, München, Germany). For in vitro and ex vivo assays, BCA was dissolved under sterile conditions in dimethylsulfoxide (DMSO) to a concentration of 50 mmol/L; for the in vivo use the concentration was 50 mg/mL. Genistein and SDG from *Linum usitatissimum* (flaxseed) were purchased from Sigma-Aldrich (Taufkirchen, Germany). Genistein was dissolved in pure DMSO under sterile conditions to a concentration of 100 mmol/L. Secoisolariciresinol diglucoside was prepared in DMSO in 30% DMSO/water under sterile conditions to a concentration of 10 mmol/L.

### Cell viability analysis and toxicity assays

Cell viability was determined using a 3(4,5 dimethylthiazol)-2,5 diphenyltetra-zolium (MTT) assay as previously described 31. Cells were plated at an appropriate density depending on the growth rate (1000–3500 cells/well) in 96-well plates 5 h prior to the drug treatment. On the fourth day, cells were incubated with MTT solution (Roth, Karlsruhe, Germany) (5 mg/mL) for 4 h at 37°C, 5% CO_2_. Cells were then lysed with 100 *μ*L isopropanol + 0.1 N HCl. The optical density of each well was determined using the microplate reader Tecan SLT spectra (Crailsheim, Germany) set to 550 nm (wavelength correction set to 690 nm) using Tecan X Fluor4 software. Plates were normally read within 1 h of adding the isopropanol. The cells without drugs were used as control. The viability of the cells was expressed as the percentage of control. Assays were performed on at least three independent experiments.

### Cell death assay and apoptosis analysis

Cell death assay was performed with propidium iodide (PI) staining purchased from Molecular Probes (Invitrogen, Darmstadt, Germany). Cells were stained for 20 min (1 *μ*g/mL) and pictures then taken with an Olympus x71 (Tokyo, Japan). Apoptosis assays were performed with modifications as described previously 32. Thus, cells plated at 12,000 cells/well in a 12-well plate and incubated for 4 days. Samples were washed with PBS (saved) and trypsinized. The pooled samples were fixed with 4% paraformaldehyde (PFA) on ice for 10 min, and then spun down at 1500 rpm for 5 min. Cells were stained with HOECHST 33258 (Invitrogen) (1 *μ*g/mL) for 10 min in darkness. The suspension was added to an objective glass and mounted on cover slips. Pictures were taken with an Olympus x71 and images with cell-F software (Olympus). Cells containing fragmented nuclei were classified as apoptotic.

### Vascular organotypic brain slice cultures

Brain slice cultures of 5-day-old Wistar rats (Charles River, Boston, MA) were prepared and maintained as previously described 33. Animals were sacrificed and the brains were removed and kept under ice-cold conditions. Frontal lobes and cerebellum were dissected of the hemispheres. The remaining brain was cut into 350 *μ*m thick horizontal slices using a vibratome (Leica VT 1000S, Bensheim, Germany). The brain slices were transferred onto ThinCert™ cell culture inserts (GreinerBioOne, Frickenhausen, Germany; pore size 0.4 *μ*m) and subsequently transferred into six-well culture dishes (GreinerBioOne) containing 1.2 mL culture medium (MEM-HBSS, 2:1, 25% horse serum, 2% l-glutamine, 2.64 mg/mL glucose, 100 U/mL penicillin, 0.1 mg/mL streptomycin, 10 *μ*g/mL insulin-transferrin-sodium selenite supplement, and 0.8 *μ*g/mL vitamin C). The slices were cultured in humidified atmosphere (35°C, 5% CO_2_). The medium was changed on the first day after preparation and from that time on every second day. The drug treatment started the day after the preparation and correlated with the medium change. Stably GFP-transfected F98 glioma cells (10,000 cells) (p-EGFP-N1 from BD Biosciences Clontech, Heidelberg, Germany) were implanted within a total volume of 0.1 *μ*L medium into the entorhinal cortex (layer II and III) 1 day after slice preparation. One day after implantation and then every second day, glioma growth and cell death were evaluated using the microscope Olympus ix71. Slice cultures were incubated with 1 *μ*g/mL PI for 20 min followed by complete medium exchange in order to visualize irreversibly damaged cells and apoptosis. Slices for pharmacology were fixed after 5 days. Slices with tumor implanted GFP-expressing F98 glioma cells were fixed after 10 days.

### Angiogenesis monitoring and vessel quantification

At the indicated culture time, slices were fixed with Immunfixative (PBS, 4% PFA, 1.2% picric acid, pH = 7.2–7.4 with NaOH) and immunostained with rabbit anti-Laminin 1:200 (Sigma-Aldrich) for vessel analysis. Vessel density quantification was performed by the overlay grid method as described 31. In brief, we designed 80 × 80 *μ*m grids over layering each image and calculated the number of vessels crossing the grids.

### In vivo study

Animal experiments were performed after the Franconian State approval and in congruence with the European Union guidelines governing the use of laboratory animals (no. 5425310806). All efforts were made to reduce animal numbers and pain suffering. Male Fisher rats weighing 190–210 g (Charles River) were deeply anesthetized by intraperitoneal injection of ketamine (Pfizer, Germany)/xylazin (Bayer Healthcare, Germany) (2:1) before fixing them in a stereotactic frame (David Kopf Instruments, Bilaney Consultants). Stably GFP-transfected F98 rat glioma cells were stereotactically implanted in a volume of 4 *μ*L (100,000 cells) with a Hamilton syringe (VWR, USA) into the right frontal lobe of the animals (2-mm lateral to bregma, 4-mm depth from dura). Drug treatment started the second day after implantation, thereafter every second day. BCA dissolved in sterile DMSO (used as vehicle) was administered through intraperitoneal injection (200 *μ*L each time) at 50 mg/kg. Tumor implant was monitored 10 days after implantation using a clinical 3 Tesla magnetic resonance imaging (MRI) scanner (Siemens Healthcare, Siemens AG, Erlangen, Germany). A modified neurological deficit assessment was documented according to four clinical scales 6. In brief, rats were clinically checked every day and evaluated according to neurological status (grade 0: normal; grade 1: tail weakness or tail paralysis; grade 2: hind leg paraparesis or hemiparesis; grade 3: hind leg paralysis or hemiparalysis; grade 4: complete paralysis (tetraplegia), moribund stage, or death). Rats were sacrificed at grade 4. Grade 1 was defined to be the onset of neurological deficit.

### MRI determination of brain edema determination

MRI was performed on a clinical 3 Tesla MR scanner (Magnetom Tim Trio Human MRI system, Siemens Healthcare, Siemens AG) with a 40-mm diameter, small field-of-view orbita surface coil as a receiver. Scout images and a 3DCISS sequence (repetition time = 9 msec, echo time = 5 msec, reconstructions with a slice thickness of 2 mm) were obtained in coronal, axial, and transverse planes to position the slices accurately. Ten coronal T1- and T2-weighted slices, each with 2-mm thickness and 0.2-mm separation (interslice gap) were then positioned on the transverse scout images to cover the tumor. T1-weighted images were acquired with a 384 × 307 matrix, field-of-view = 70 × 70 mm, repetition time = 507 msec, echo time = 17 msec, and a total scan time of 3 min 42 sec. For contrast-enhanced images, each animal received 0.1 mL per kg body weight of contrast agent (Gadovist 1.0; Bayer Pharma, Leverkusen, Germany) i.p. 10 min prior to the acquisition of T1-weighted sequences. The T2-weighted images were acquired with a 320 × 256 matrix, field-of-view = 91 × 91 mm, repetition time = 4500 msec, echo time = 158 msec, and a total scan time of 6 min 12 sec. Imaging analysis was performed for each rat using Osirix (GNU General Public License built-in image processing software) to outline tumor volume on the T1-weighted contrast-enhanced images. Total tumor volume was calculated as the summed area on all slices, multiplied by the slice separation, and compared to histology-derived tumor volume. Additionally, edema volume was measured by subtracting the tumor volumes as derived from T1 and T2 tumor volumes derived from the, respectively, weighted images.

### Immunofluorescence

The rat brains of the in vivo study were fixed in 4% PFA and thereafter incubated in 20% sucrose for 3 days. The brains were frozen in liquid nitrogen and embedded in Tissue Tek. Cryosections (10 *μ*m) were stained with rat anti-RECA-1 at 1:500 (Abd Serotec, Puchheim, Germany). Detection of bounded primary antibodies was accomplished with Alexa Fluor 568 goat anti-rabbit IgG secondary antibody and Alexa Fluor 568 goat anti-rat IgG secondary antibody (Molecular Probes, Life Technologies, Darmstadt, Germany), respectively, both at 1:1000. The cryosections were counterstained with HOECHST 33258 (Invitrogen, Life Technologies) at a concentration of 1 *μ*g/mL.

### Statistical analysis

Analysis was performed using unpaired two-sided Student's *t-*test (MS Excel). Data from cell viability analysis and toxicity assays were obtained from at least four independent experiments. The level of significance was set at *P < 0.05. Error bars represent ±SD. For survival analysis of the in vivo study we used the GraphPad Prism software (GraphPad Software, Inc., La Jolla). Cell death intensity was quantified by *Mac Biophotonics-Image J* and vessel density through the overlay grid method 34 by Adobe Photoshop (Adobe Photoshop Inc., San Jose, CA).

## Results

### Various isoflavonoids with different toxicity profiles on glioma cell growth

**Figure 1:**
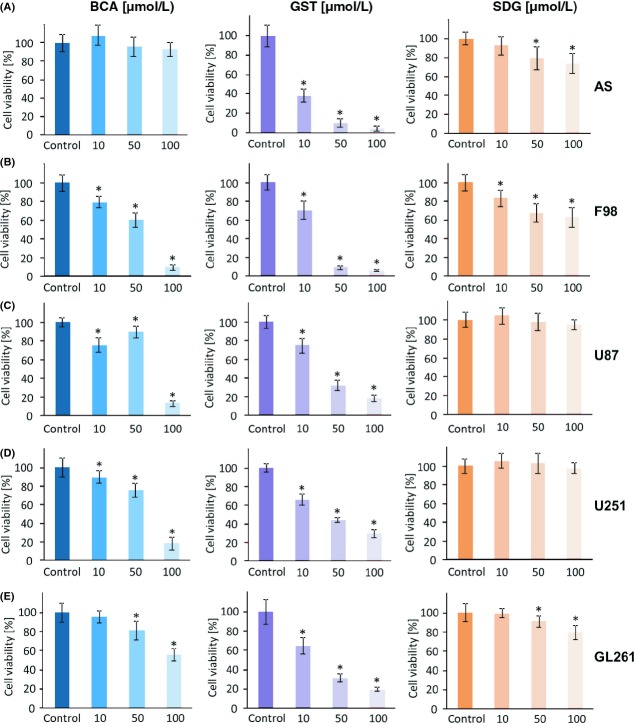
Isoflavonoids impede astrocytes and malignant glioma cell growth with differential efficiency. (A) Primary rodent astrocytes (AS) were treated with various concentrations of biochanin A (BCA), genistein (GST), and secoisolariciresinol diglucoside (SDG) and cell survival was monitored. BCA is nontoxic for primary astrocytes even at high concentrations. GST induces astrocyte cell death at low concentration. SDG is toxic to astrocytes from 50 *μ*mol/L onward. (B) Rodent malignant gliomas (F98) were treated with various concentrations of BCA, GST, and SDG and glioma cell survival was monitored. All three isoflavonoids are toxic already at low concentrations. (C, D) Human malignant gliomas (C, U87; D, U251) were treated with various concentrations of BCA, GST, and SDG and glioma cell survival was monitored. Note that SDG does not affect human glioma growth. BCA and GST causes cell death. (E) Murine malignant gliomas (GL261) were treated with various concentrations of BCA, GST, and SDG and glioma cell viability was measured. Differences were considered statistically significant with values mean ± SD (*n* ≥ 3 per group; unpaired two-sided *t*-test, *P* < 0.05).

In order to investigate whether isoflavonoids are generally toxic to normal differentiated brain cells, we first established the toxicity profile of various isoflavonoids on rat primary astrocytes (Fig.1A). BCA showed no toxicity toward primary astrocytes within a wide concentration range. At maximum concentrations, BCA reduced growth of primary astrocytes to about 5% compared to control conditions (Fig.1A, left). Both isoflavonoids GST and SDG tested within the same concentration assays showed a significant reduction in cell viability already at 10 *μ*mol/L. The decrease in cell viability was pronounced in the case of GST, with over 60% of cells dying at 10 *μ*mol/L GST. At 100 *μ*mol/L GST reduced cell survival to below 5% (Fig.1A, middle). SDG appeared less toxic, with significant decrease in cell survival to 80–85% at 50 *μ*mol/L and 100 *μ*mol/L, respectively (Fig.1A, right).

**Figure 2:**
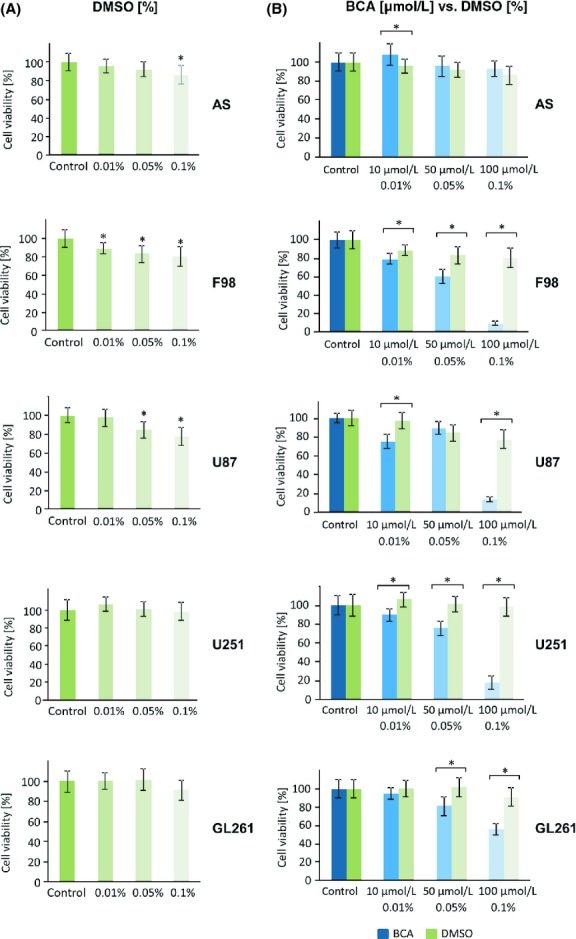
Comparative analysis of the solvent dimethylsulfoxide (DMSO) and BCA on malignant glioma cell growth. (A) Primary rodent astrocytes (AS), rodent glioma cells (F98), human glioma cells (U87, U251), and murine glioma cells (GL261) were treated with various concentrations of the solvent DMSO and cell survival was monitored. Note that a high concentration of DMSO at 0.1% (vol/vol) reduces cell growth in some cells. (B) BCA (blue bars) DMSO (green bars) solvent-matched analysis. The facilitated BCA concentration is given and compared to the respective final DMSO concentration. Differences were considered statistically significant with values mean ± SD (*n* ≥ 3 per group; unpaired two-sided *t*-test, *P* < 0.05).

We next tested the effect of these isoflavonoids on established glioma cells, first on F98 rat glioma cells (Fig.1B). GST was more potent here in inducing cell death than BCA, especially at 50 *μ*mol/L (Fig.1B, left, middle). SDG appeared to be less effective in comparison to BCA and GST (Fig.1B, right). In human glioma cells U87 (Fig.1C) and U251 (Fig.1D), the isoflavonoid BCA showed significant reduction in cell growth of down to 10% in comparison to the controls (Fig.1C and D, middle). It was interesting to note that SDG did not affect cell proliferation of human U87 and U251 glioma cells, which grew equally well in comparison to untreated controls (Fig.1C and D, right). We also tested these isoflavonoids on the established murine GL261 glioma cells (Fig.1E). Although BCA (Fig.1E, left) and SDG (Fig.1E, right) were both effective in reducing GL261 cell growth from 50 *μ*mol/L onward, BCA showed a broader efficacy in all other tested glioma cell lines. GST reduced the cell viability of GL261 40–80% (Fig.1E, middle) and had the highest potency of the tested isoflavonoids. The growth inhibitory effect was observed on all cells independently of their malignant status already at the lowest concentration (10 *μ*mol/L) (Fig.1A–E, middle). Since BCA is solved in DMSO, we further tested the effects of DMSO on astrocytes and glioma cell proliferation (Fig.2). DMSO appeared to reduce cell viability at high concentrations in astrocytes, F98 and U87 glioma cells (Fig.2A). Next, we compared the cell viability of BCA treatment with the respective DMSO-matched controls. These results confirmed our initial findings that BCA is gliomatoxic and has no damaging effects on primary astrocytes (Fig.2B).

### Biochanin A induces apoptotic cell death in human glioma cells

**Figure 3:**
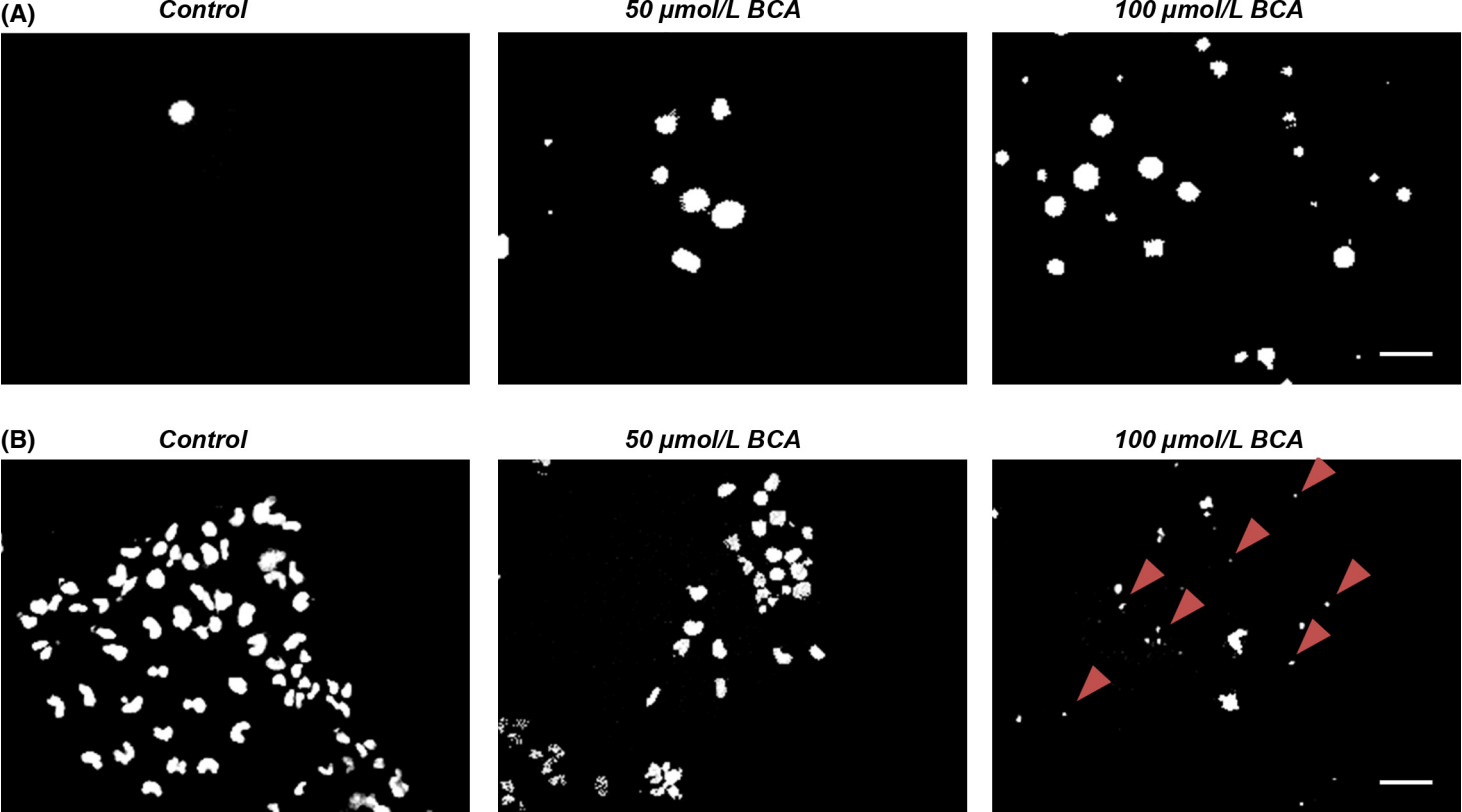
Biochanin A induces apoptosis in human glioma cells. (A) Human glioma (U251) cell death after BCA treatment was monitored in vivo by propidium iodide (PI). Dead cells take up the PI dye and appear as white-stained nuclei. Scale bar represents 100 *μ*m. (B) Apoptosis of human gliomas (U251) monitored by HOECHST dye. Fragmented nuclei are accumulating after BCA treatment (arrow heads). Scale bar represents 50 *μ*m.

The results of the BCA treatment could be further confirmed by monitoring cell death with PI staining in human U251 glioma cells (Fig.3A). With increasing concentrations, BCA-treated gliomas exhibited higher numbers of dead cells. The apoptosis assay on BCA-treated glioma cells also supported the cell viability results. The extent of apoptosis was evaluated by morphological analysis of the nuclei. Fragmented nuclei were found in U251-treated cells stained for nuclei with Hoechst 33342, which were assessed as an indicator for apoptosis (Fig.3B). The dose–response analysis of BCA on glioma cells could be confirmed by investigating apoptotic cell death (Fig.3B).

### Biochanin A operates neuroprotective in an organotypic brain environment

**Figure 4:**
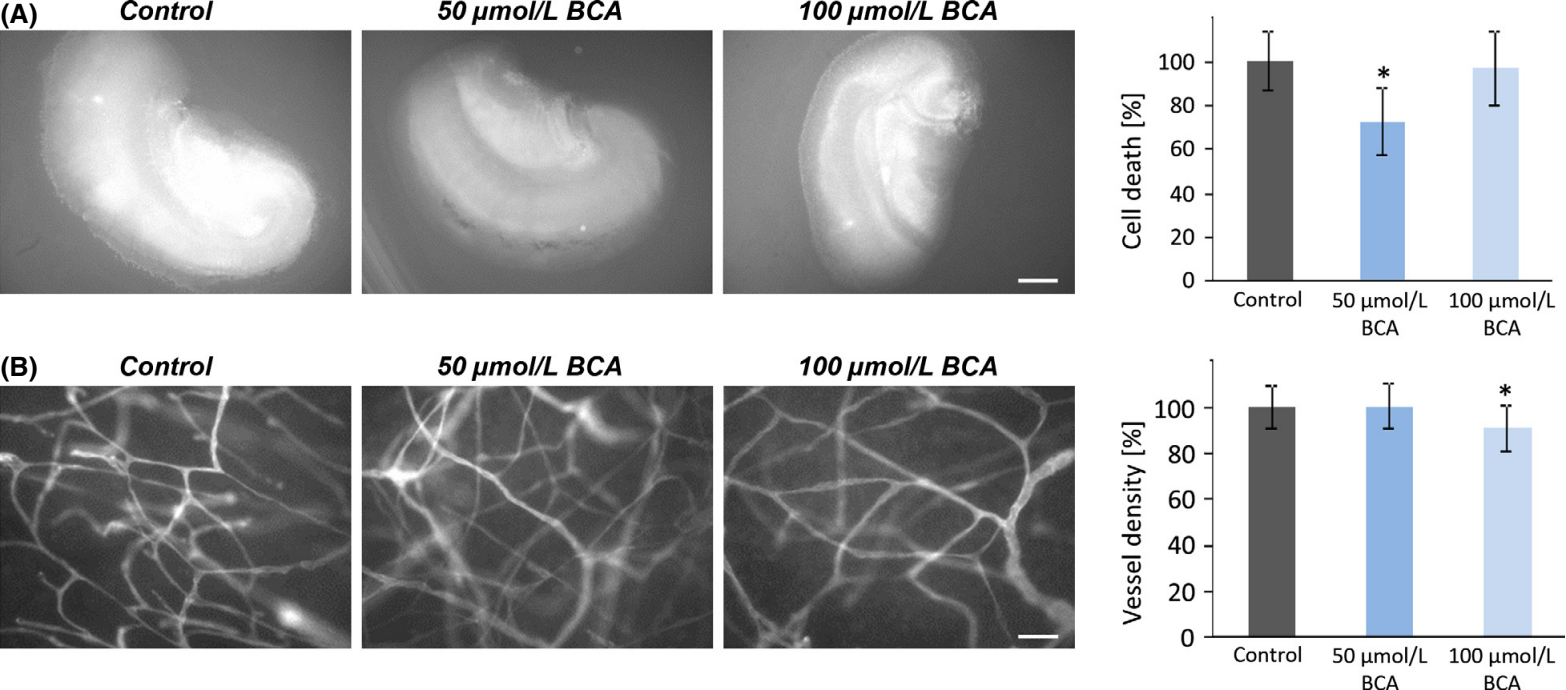
Biochanin A is neuro- and vasculoprotective in organotypic brain environment. (A) BCA treatment showed neuronal protective effects in rat native brain slices. After 5 days culture, cell death was evaluated (propidium iodide). A quantity of 50 *μ*mol/L BCA showed a significant decrease in cell death. Scale bar represents 1 mm. Differences were considered statistically significant with values mean ± SD (*P < 0.01, unpaired two-sided *t*-test, *n* ≥ 14 per group). (B) Vessel density was significantly reduced at 100 *μ*mol/L BCA treatment. Scale bar represents 50 *μ*m. Differences were considered statistically significant with values mean ± SD (*P < 0.01, unpaired two-sided *t*-test, *n* ≥ 10 per group).

We next tested whether BCA affects neuronal survival and integrity. For this native, rat brain slices were treated with the concentration of BCA already tested in the cell viability assays (Fig.4). Advantage of this ex vivo model is that it enables pharmacological testing in real-time mode 33. Brain sections were cut and slices were cultured on permeable PET membranes bathed in culture medium. Treatment was performed by adding BCA into the medium and cell death was assessed with PI after 5 days in culture. Quantitative results showed a significant cell death decrease by 30% at 50 *μ*mol/L BCA (Fig.4A). The slices were then fixed and stained for laminin and subsequently vessel density was determined by the overlay grid method 34. We found that the vascular density decreased by 15% in 100 *μ*mol/L BCA-treated brain slices compared to non-treated brain tissues (Fig.4B). We conclude that BCA acts neuroprotective and also vasculoprotective within an organotypic brain environment.

### Biochanin A reduces glioma tumor growth and exhibits antiangiogenic properties

**Figure 5:**
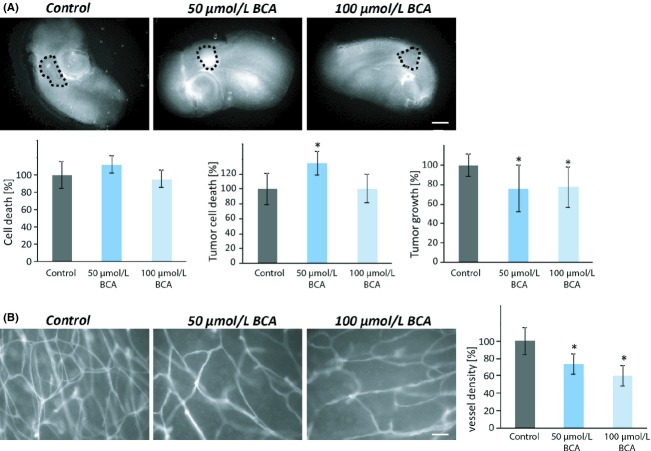
Biochanin A alleviates tumor growth and protects neurons in an organotypic brain environment. (A) 50 *μ*mol/L BCA treatment induces tumor cells death in glioma-implanted brain slices. The dashed circle marks the tumor from the normal tissue. The normal tissue shows no cell death in the BCA-treated groups. F98 GFP expressing glioma cells were implanted in brain slices and after 8 days, cell death was evaluated. BCA reduces tumor growth. Differences were considered statistically significant with values mean ± SD (*P < 0.05, unpaired two-sided *t*-test, *n* ≥ 20). Scale bar represents 1 mm. (B) After 10 days in culture, tumor-implanted slices were fixed and stained for laminin. Vessel density in peritumoral area was obviously reduced after BCA treatment. Differences were considered statistically significant with values mean ± SD (*P < 0.01, unpaired two-sided *t*-test, *n* ≥ 10 per group). Scale bar represents 50 *μ*m.

We next implanted glioma cells into normal brain tissue and monitored tumor growth and brain tissue integrity. Glioma tumors showed invasive cell growth and destructive effects on its host environment (Fig.5). After 8 days in culture, cell death was measured with PI in both the normal brain tissue in the control group and the BCA-treated group. There was no visible difference (Fig.5A, left). In analyzing the effects of BCA on treatment with 50 *μ*mol/L revealed enhanced tumor cell death. In this context, BCA acted as a glioma-toxic agent (Fig.5A, middle). Subsequent investigations of tumor growth over time confirmed the initial findings in single-cell cultures that BCA is glioma toxic in a concentration-dependent manner. Concentrations of 50 *μ*mol/L BCA reduced tumor growth up to 25%, which could not be increased by higher concentrations of BCA (Fig.5A, right). After 10 days in culture, tumor-implanted brain slices were fixed and stained for laminin. Noteworthy, vessel density in peritumoral areas was visibly reduced following BCA treatment. Quantitative results verified these findings, showing a decrease by 30–40% compared to controls (Fig.5B).

### Biochanin A reduces tumor-related cerebral edema and alleviates clinical deterioration in vivo

**Figure 6:**
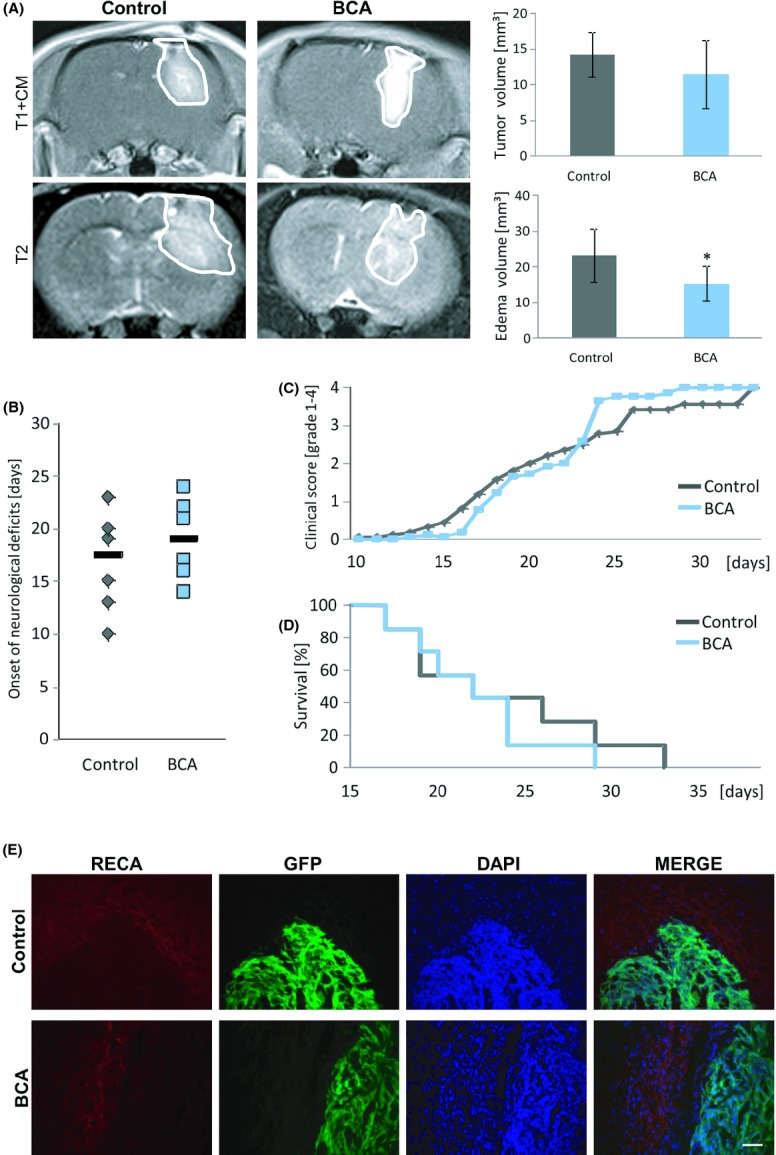
Biochanin A reduces brain tumor-induced edema and alleviates clinical deterioration in vivo. (A) Representative MR images 9 days after tumor implantation in male Fisher rats of control and BCA-treated gliomas. The tumor bulk (marked with continuous white line) was visualized after application of intraperitoneal contrast agent and subsequent T1-weighted imaging (T1 + CM) and corresponding T2-weighted images of brains from control and BCA-treated gliomas. The marked area indicates total tumor volume (including peritumoral and edema zone). Tumor volume was quantified from T1-weighted MR images. The edema zone was quantified by T2- and T1-weighted MR images. Tumor volume in the BCA-treated rats tends to be smaller in comparison to the control group. The edema zone is significantly smaller in the BCA-treated group compared to the control group. (B) Animals were clinically assessed on a daily basis according to their neurological status (grade 0: normal; grade 1: tail weakness or tail paralysis; grade 2: hind leg paraparesis or hemiparesis; grade 3: hind leg paralysis or hemiparalysis; grade 4: complete paralysis (tetraplegia), moribund stage or death). Therefore, the onsets of neurological symptoms were measured. Animals with BCA treatment tends to develop later neurological deficits compared to control animals. (C) Clinical progression of neurological deficits of the BCA-treated group and the control group. BCA group tends to have less neurological deficits in the first 20 days after tumor implantation. (D) Kaplan–Meier survival curves of BCA-treated animals and the control animals. Statistical significance was calculated with Student's *t*-test (*n* = 7 for the BCA group; *n* = 8 for the control group). (E) Representative brain images of BCA-treated rats and control group both groups bearing GFP-expressing F98 glioma cells (green) stained with the vascular endothelial cell marker RECA (red). Scale bar represents 50 *μ*m.

Since BCA appeared to be effective as an antitumor angiogenic and neuroprotective agent, we investigated this isoflavonoid in vivo. For this, we stereotactically implanted glioma cells into rat brains and monitored tumor growth by MRI scans (Fig.6). Nine days after implantation, in vivo tumor monitoring by T1-weighted MRI scans revealed that BCA-treated gliomas tended to be smaller than the control tumor group (Fig.6A). Three Tesla contrast agent-enhanced T1-weighted MRI scans further demonstrated reduced perifocal cerebral edema in animals treated with intraperitoneal BCA (Fig.6A). In the assessment of onset of clinical signs after tumor implantation, BCA-treated animals developed neurological deficits later than control-treated animals (Fig.6B). Monitoring the clinical progression of neurological deficits in animals with BCA treatment showed that they exhibited fewer neurological deficits in the first 20 days after tumor implantation (Fig.6C). Although the difference was not statistically significant, the Kaplan–Meier survival analysis showed that BCA-treated animals tended to survive longer than controls (Fig.6D).

We next investigated tumor angiogenesis in vivo by comparing the vasculature of BCA-treated animals with that of the control group. Staining for the vascular endothelial cell marker RECA revealed no obvious differences between these groups (Fig.6E). Altogether, our study revealed that BCA treatment can alleviate tumor-induced brain edema in vivo.

## Discussion

In this study, we investigated the impact of isoflavonoids on glioma morphology and function. Our results demonstrate that BCA in particular can reduce the oncogenic progression of glioma cells. Investigation of different isoflavonoids in identical conditions revealed that they exert different toxic and efficacy profiles. Our study shows that BCA significantly affects malignant gliomas while exhibiting a low toxic profile toward nonmalignant astrocytes. Having studied the effects of various isoflavonoids in the context of cancer cells, our investigations showed that the toxicity profile of BCA, GST, and SDG differ profoundly. Although GST is very effective in inducing glioma cell death, it is highly toxic for primary astrocytes. Prior reports showed on the contrary that GST slightly reduces human astrocyte viability within 24 h and led to increased reactive oxygen species formation 21. Our study, however, included analysis over an extended time period of 4 days within this timeframe GST and SDG exhibited toxicity toward primary astrocytes. In the identical setting, however, cell growth and viability of primary astrocytes were not affected toward BCA treatment. Since BCA and GST both share a comparable chemical structure what makes the differences in toxicity between these two compounds? Biochanin A or 4-methylgenistein is an O-methylated version of GST with different biological properties. First, BCA has been shown to function as a ligand for the human aryl hydrocarbon receptor 35. This is of particular relevance since the human aryl hydrocarbon receptor is implicated in various processes such as transformation, tumor genesis, and inflammation 36. A similar binding on this receptor has not been shown for GST. Furthermore, comparative gene expression analysis of BCA and GST in breast cancer cells (MCF7) and nontransformed breast cells revealed that gene expression challenges were mostly beneficial, involving induction of tumor suppressor genes in BCA-treated cancer cells 37. In addition, it has been shown that the metabolic pathway in mammalian cells differs significantly between BCA and GST making BCA nontoxic to nontransformed cells 38.

More important was the finding that BCA, besides its low toxic profile toward healthy brain tissue, exerted neuroprotective effects and hence prevented spontaneous neuronal damage. This is of particular relevance since neuronal damage and neurodegeneration are major features of primary malignant-brain tumors 6,8, which can lead to tumor-associated epileptic seizures 39. Tumor-induced neurodegeneration is also associated with the development of tumor-induced cerebral edema 7,40, the uncontrolled increase of which is a major cause of morbidity and death in gliomas due to cerebral herniation 10,40,41. Previously it has been shown that reduction in tumor-induced neurodegeneration can reduce tumor-induced brain swelling. Thus, it is tempting to speculate whether the neuroprotective action of BCA contributes to alleviated brain edema. Notably, an alternative mechanism is the vasogenic impact also known to induce tumor-induced edema. By its antiangiogenic action, BCA may operate toward a vascular stabilizing environment thereby alleviating vasogenic brain edema.

Our ex vivo and in vivo investigations revealed that BCA exhibits antiedematous and antiangiogenic properties. BCA decreased tumor-associated edema and delayed the onset of clinical deterioration. These results indicate that BCA exhibits various toxicity profiles and can be beneficial as a potential substitute to established chemotherapeutic agents. Caloric restriction is often performed by dietary components such as isoflavonoids constituting an integral part of the diet 17,19. Whether the reported beneficial effects of caloric restriction can be traced back to the effects of BCA remains to be shown 13–16. Since the development of tumor-induced edema has two pathogenic components, that is, a cytotoxic and vasogenic, it remains a matter of speculation as to which mechanism primarily contributes to the BCA-associated effects like normalization of tumor vessels and reduction in tumor-induced cell death. Nevertheless, the management of cerebral edema 42, currently comprising primarily of treatment with dexamethasone 11, represents a major component of the battle against brain-tumor associated comorbidities. Thus, BCA may provide a dietary therapeutic approach with beneficial and supportive effects for the management of tumor-induced brain edema.

## Conclusion

In summary, our results show that dietary isoflavonoids differ in their impact on glioma cells. BCA in particular is vasculo- and neuroprotective, and can alleviate tumor-induced brain edema. These data further indicate that BCA can synergistically support multimodal therapies with a low toxic profile. However, BCA alone as a monotherapy does not seem to be effective, at least in case of intraperitoneal administration.
